# Mitigation strategies to safely conduct HIV treatment research in the context of COVID‐19

**DOI:** 10.1002/jia2.25882

**Published:** 2022-02-09

**Authors:** Merle Henderson, Sarah Fidler, Beatriz Mothe, Beatriz Grinsztejn, Bridget Haire, Simon Collins, Jillian S. Y. Lau, Maureen Luba, Ian Sanne, Roger Tatoud, Steve Deeks, Sharon R. Lewin

**Affiliations:** ^1^ Department of Infectious Disease Imperial College London London UK; ^2^ IrsiCaixa AIDS Research Institute HUGTIP Badalona Spain; ^3^ Fundação Oswaldo Cruz Rio de Janeiro Brazil; ^4^ Kirby Institute Sydney New South Wales Australia; ^5^ i‐Base London UK; ^6^ The Peter Doherty Institute for Infection and Immunity The University of Melbourne and Royal Melbourne Hospital Melbourne Victoria Australia; ^7^ AVAC Lilongwe Malawi; ^8^ University of the Witwatersrand Johannesburg South Africa; ^9^ International AIDS Society Geneva Switzerland; ^10^ University of California, San Francisco (UCSF) San Francisco California USA

**Keywords:** HIV, clinical trial, analytical treatment interruption (ATI), COVID‐19, SARS‐CoV‐2, risk mitigation

## Abstract

**Introduction:**

The International AIDS Society convened a multidisciplinary committee of experts in December 2020 to provide guidance and key considerations for the safe and ethical management of clinical trials involving people living with HIV (PLWH) during the SARS‐CoV‐2 pandemic. This consultation did not discuss guidance for the design of prevention studies for people at risk of HIV acquisition, nor for the programmatic delivery of antiretroviral therapy (ART).

**Discussion:**

There is strong ambition to continue with HIV research from both PLWH and the research community despite the ongoing SARS‐CoV‐2 pandemic. How to do this safely and justly remains a critical debate. The SARS‐CoV‐2 pandemic continues to be highly dynamic. It is expected that with the emergence of effective SARS‐CoV‐2 prevention and treatment strategies, the risk to PLWH in clinical trials will decline over time. However, with the emergence of more contagious and potentially pathogenic SARS‐CoV‐2 variants, the effectiveness of current prevention and treatment strategies may be compromised. Uncertainty exists about how equally SARS‐CoV‐2 prevention and treatment strategies will be available globally, particularly for marginalized populations, many of whom are at high risk of reduced access to ART and/or HIV disease progression. All of these factors must be taken into account when deciding on the feasibility and safety of developing and implementing HIV research.

**Conclusions:**

It can be assumed for the foreseeable future that SARS‐CoV‐2 will persist and continue to pose challenges to conducting clinical research in PLWH. Guidelines regarding how best to implement HIV treatment studies will evolve accordingly. The risks and benefits of performing an HIV clinical trial must be carefully evaluated in the local context on an ongoing basis. With this document, we hope to provide a broad guidance that should remain viable and relevant even as the nature of the pandemic continues to develop.

## 1 INTRODUCTION

In January 2020, a novel coronavirus, the severe acute respiratory syndrome coronavirus 2 (SARS‐CoV‐2), was identified as the causative agent of COVID‐19 disease [1]. The World Health Organization (WHO) declared SARS‐CoV‐2 infection a pandemic on 11 March 2020 [2]. As of December 2021, there have been an estimated 280 million confirmed cases of COVID‐19, including over 5.4 million deaths globally [3], with an estimated infection fatality ratio approaching 1.15% (0.78–1.79 95% CI) [3, 4, 5]. Infection with the alpha variant of SARS‐CoV‐2 may also have a higher risk of mortality (mortality hazard ratio 1.64 [1.32–2.04 95% CI]) [6]. Evidence suggests that there is a higher risk of infection with the Delta variant [7, 8, 9, 10], and the rapid spread of the Omicron variant, which was first reported in South Africa in November 2021 and has since been identified in multiple other countries, raises significant concerns for its increased transmissibility [11, 12].

The COVID‐19 pandemic has had a significant impact on people living with HIV (PLWH) as a result of the global disruption to HIV services and programs [13]. Many risk factors for severe COVID‐19 are over‐represented among PLWH. Severe COVID‐19 has been associated with older age (>60 years), male sex and the presence of co‐morbidities (such as cardiovascular disease, renal disease, hypertension, diabetes, obesity, ongoing cancer and recent organ recipients) [14]. Other important risk factors for more severe disease are poverty, poor housing and being a member of ethnic minority populations [14].

Evidence suggests that PLWH on successful antiretroviral therapy (ART) may be at an increased risk of COVID‐19 acquisition, although the interplay of socio‐economic status and therefore exposure may confound these studies [15, 16]. While some studies show that PLWH are not at increased risk of more severe COVID‐19 [16], others report that this is not always the case [15, 17, 18]. A systematic review of 22 articles found that HIV may be a risk factor for SARS‐CoV‐2 acquisition and severe COVID‐19, but residual cofounding of co‐morbidities could not be excluded [19, 20, 21, 22]. A WHO report, based on surveillance data from 37 countries between 01 January 2020 and 29 April 2021, demonstrated that PLWH hospitalized with COVID‐19 had an increased risk of severe disease and in‐hospital mortality than people without HIV [23]. For PLWH with severe immunosuppression or uncontrolled HIV viraemia, fatality rates for COVID‐19 remain uncertain [19, 20, 24, 25], although recent evidence suggests that these individuals have a higher risk of severe COVID‐19 disease [26, 27]. Similarly, a higher risk of complications from COVID‐19 has been reported among PLWH with a low CD4^+^ T cell count nadir prior to ART initiation compared to those with higher nadirs [28].

While SARS‐CoV‐2 vaccines provide only limited protection against acquisition and transmission of infection [29], and evidence suggests that immunity may wane over time, vaccination has been shown to reduce the severity of COVID‐19 disease [30]. SARS‐CoV‐2 vaccines have been proven to be safe and effective in PLWH [31, 32, 33], and the WHO has recommended that all PLWH should be prioritized for early vaccination [34, 35]. However, immunogenicity studies suggest that there may be some differences between HIV‐positive and HIV‐negative vaccine recipients [36]. Recent findings of improved immune responses following a third dose of an mRNA vaccine in solid organ transplant recipients [37, 38] led to the CDC and WHO recommending a third dose as part of the primary vaccination schedule for people who have moderate to severe immunocompromise, including people with advanced or untreated HIV, and further “booster” doses for all PLWH [39, 40, 41]. The recent emergence of the Omicron variant has led to an upscale in vaccination booster dose programmes [42], and the likely global endemic future of COVID‐19 suggests that booster doses may be required long term.

## 2 DISCUSSION

The SARS‐CoV‐2 global burden, challenges and responses have been remarkable, both in financial and human terms. However, this has also significantly disrupted ongoing and planned HIV clinical research efforts [43, 44], with most trials being paused between March 2020 into 2021. As a result, an international steering group was virtually assembled by the International AIDS Society (IAS, see acknowledgements) to determine key questions for discussion with regard to how to safely and justly resume HIV clinical research in the context of ongoing SARS‐CoV‐2 transmission. Two virtual consultations were held on 9 and 17 December 2020. Thirty four participants were identified for inclusion, of which 22 were in attendance. A summary document was subsequently circulated for comments to a group of 40 international stakeholders. A position statement was drafted and approved by all participants, with an update on 6 October 2021 based on emerging evidence. The main themes that formed part of both discussion meetings were reported and informed the outcomes of the consultation [45].

All recommendations are based on current evidence. The rapidly changing landscape of the COVID‐19 pandemic is acknowledged and if substantial changes in the pandemic emerge, the panel will be reassembled to further update the guidelines.

A high‐level summary of recommendations is presented in Table 1.

**Table 1:** High‐level summary of recommendations

1.1 Balancing risks and benefits of HIV research in the context of COVID‐19 Local community SARS‐CoV‐2 incidence levels and mobility restrictions should be considered before HIV study enrolment. If local infection rates increase, pausing a study that requires in person study visits may be necessary. Those at high risk of severe COVID‐19 disease, including the presence of co‐morbidities, advanced or untreated HIV, should have specific counselling prior to study enrolment to emphasize two main considerations: the increased risk of acquiring SARS‐CoV‐2 infection associated with study visits and intervention; and of the increased of severe COVID‐19 disease related to co‐morbidities.
1.2 Balancing risks and benefits of HIV research in the context of COVID‐19 Local community SARS‐CoV‐2 incidence levels and mobility restrictions should be considered before HIV study enrolment. If local infection rates increase, pausing a study that requires in person study visits may be necessary. Those at high risk of severe COVID‐19 disease, including the presence of co‐morbidities, advanced or untreated HIV, should have specific counselling prior to study enrolment to emphasize two main considerations: the increased risk of acquiring SARS‐CoV‐2 infection associated with study visits and intervention; and of the increased of severe COVID‐19 disease related to co‐morbidities.
1.3 The impact of COVID‐19 prophylactic vaccines on HIV trials Access to a WHO‐approved SARS‐CoV‐2 vaccine is recommended before recruitment into an HIV study. Where a vaccine is routinely available, and based on the most up‐to‐date booster vaccination advice, deferral of trial entry is recommended until 4 weeks after the completion of the vaccination series, when optimal immunogenicity and protection are anticipated. This is particularly relevant for trials that include immunotherapies and/or analytical antiretroviral treatment interruptions (ATIs). If a SARS‐CoV‐2 vaccine becomes available during the trial, then whenever possible, immunotherapy that could potentially interfere with an optimal vaccine response or the initiation of an ATI should be deferred until 4 weeks after the last vaccine dose. The vaccine should be offered to any study participants already enrolled into the protocol. Careful evaluation must be applied to any study of interventions that could compromise immune function (e.g. immune activators, immune modulators, latency‐reversing agents and therapeutic HIV vaccines) and that could adversely impact the natural history of COVID‐19 and/or response to a COVID‐19 vaccine.
1.4 Ethics regarding equitable access to vaccination and vaccination hesitancy There is a lack of equitable access to effective COVID‐19 vaccines globally. Where a SARS‐CoV‐2‐approved vaccine is not routinely available, it is recommended that the HIV trial investigators offer vaccination prior to study enrolment, in particular for trials that could compromise immune function and/or include an ATI. For those refusing vaccination, the level of risk related to the study and of severe COVID‐19 disease must be carefully considered by all study stakeholders prior to enrolment.
1.5 Study implementation and management The risk of SARS‐CoV‐2 acquisition should be mitigated by: (1) the provision of personal protective equipment (PPE) for study participants, all research and clinical staff and community partners as needed; (2) limitation of the frequency of in‐person study visits where feasible; and (3) the use of remote study visits (telemedicine), electronic consent and study records where feasible and permissible. Exclusion of active SARS‐CoV‐2 infection through the offer of SARS‐CoV‐2 PCR testing is recommended, where available, before enrolment, at least 72 hours prior to any investigational drug dosing and ATI and at regular intervals throughout the trial period. In some settings, frequent antigen testing for all study participants may be acceptable, dependent on the study complexity and if an ATI is involved. Frequency of SARS‐CoV‐2 testing will be dependent on local incidence patterns. For potential participants who test positive for SARS‐CoV‐2 at enrolment, deferral of study enrolment is recommended until proven SARS‐CoV‐2 negative or no longer deemed infectious, based on local guidelines. If local guidelines are not available, consider reference to WHO technical guidance on COVID‐19 [46]. A negative SARS‐CoV‐2 PCR test result should be confirmed immediately prior to any planned analytical antiretroviral treatment interruptions (ATIs) or the administration of any immunotherapy that might conceivably put a person at high risk of COVID‐related morbidity.

### 2.1 Balancing risk and benefits of HIV research in the context of COVID‐19

It is important to consider the risks and benefits of conducting versus deferring HIV clinical trials in PLWH during surges of SARS‐CoV‐2 infections within a community. As it is likely that SARS‐CoV‐2 infection will become endemic and persist in some manner for years, the consensus reached was that the development of novel treatment and cure strategies for HIV must continue.

To determine if HIV trials are safe to resume or open, we recommend that each study is reviewed and evaluated on a case‐by‐case basis, dependent on the type of intervention planned, background prevalence and incidence of SARS‐CoV‐2, local hospital capacity and availability of staffing, as well as access to SARS‐CoV‐2 treatment and uptake of a safe and effective preventative vaccine. It is acknowledged that there is not equitable access to effective treatment and prophylaxis against SARS‐CoV‐2 infection [47] and, in their absence, it may not be appropriate to conduct certain trials, such as those involving an analytical treatment interruption (ATI). In accordance with international clinical trials delivery guidance, we recommend that all study stakeholders carefully consider the potential SARS‐CoV‐2 risks with the study participants [48, 49].

Each clinical trial should have a current COVID‐19 risk mitigation plan, clearly outlined in all study‐related material and participant information sheets, including a plan for COVID‐19 vaccines. The COVID‐19 issues will require robust informed consent to ensure that study participants are aware of the implications of acquiring COVID‐19 during the research. The balance of the preservation of trial scientific integrity in order to retain the capacity to generate meaningful data versus COVID‐19 risk mitigation to ensure the safety and wellbeing of study participants is key.

### 2.2 The impact of COVID‐19 prophylactic vaccines on HIV trials

SARS‐CoV‐2 vaccines are currently available under either full approval or emergency use. These include mRNA, viral vector, protein and killed vaccines. Early vaccine studies included a limited number of PLWH, for example in a phase 3 study of an adjuvanted protein vaccine (Novavax) in South Africa, 6% of participants were PLWH [50]. Current recommendations are for PLWH on ART to be fully vaccinated against SARS‐CoV‐2 [34], with no specific preference for an individual vaccine, although this may well change as further data emerge.

#### 2.2.1 Access and equity to SARS‐CoV‐2 vaccines

As the number of administered prophylactic SARS‐CoV‐2 vaccine doses continues to grow daily [3], there remains a stark inequality in access to vaccination worldwide. As of December 2021, only 9.5% of the population in low‐income countries had received at least one dose of a preventative vaccine versus 66.7% in high‐income countries [47]. While international efforts aim to increase vaccination coverage worldwide, through the COVAX alliance and vaccine donations, projections suggest that many countries may lack substantial levels of vaccination until 2023 [51]. There are major disparities in who acquires SARS‐CoV‐2 and disease severity once infected, as well as people's ability to access treatment and prevention for COVID‐19. Given that many of these same trends exist in PLWH, the overall burden of SARS‐CoV‐2 will likely prove to be much higher globally on those with HIV than those without HIV.

#### 2.2.2 Emerging SARS‐CoV‐2 variants

As the pandemic has continued to evolve and new SARS‐CoV‐2 variants with increased transmissibility have emerged, concerns have been raised about the sustained effectiveness of current vaccines [30, 52]. Data are emerging discussing the efficacy of available vaccines against the new variants [30, 53, 54, 55, 56]. New evidence suggests that immunity from previous infection or two vaccine doses without a booster vaccination may not be effective in preventing infection with the Omicron variant [57]. Vaccine availability and local variants, therefore, must be considered if resuming or opening clinical research.

#### 2.2.3 Timing of vaccines during the conduct of a clinical trial

Timing of vaccination must be considered prior to resuming clinical research in PLWH. Ensuring the safety and wellbeing of study participants and staff while maintaining maximal scientific integrity is key.

Given the experimental nature of interventions, specifically in cure research, and the potential adverse effects of an ATI, we recommend here a longer separation between intervention and/or ATI and receipt of a COVID‐19 vaccine of 4 weeks. Hence, for all HIV ATI interventional or immune modulatory studies, the consensus recommendation is to delay ATI until at least 4 weeks post receipt of an approved SARS‐CoV‐2 vaccine. If SARS‐CoV‐2 vaccination is not available through national health systems, the study team should consider vaccine provision prior to ATI. This is to ensure adequate protection against severe COVID‐19 disease and avoid any confounding of immunogenic effects from the vaccine with the intervention in question or the risk of inducing viral replication during an ATI. However, should SARS‐CoV‐2 booster dosing become recommended during an HIV trial protocol, the study protocol should be modified to allow for vaccination. Additionally, influenza vaccination should also be offered to all PLWH study participants in particular, for studies including an ATI or immunomodulatory treatments. Guidelines currently recommend co‐administration of SARS‐CoV‐2 and influenza vaccines [58].

#### 2.2.4 Novel SARS‐CoV‐2 treatments

The field of SARS‐CoV‐2 treatment is rapidly evolving and new antiviral agents are increasingly being identified and approved for use as treatment for SARS‐CoV‐2 [59, 60]. The consideration of drug–drug interactions for ART and any additional HIV cure strategy must be carefully evaluated in the context of enrolment into novel SARS‐COV‐2 treatment trials.

### 2.3 Ethics of proceeding with those not willing to receive a COVID‐19 vaccine

Vaccination hesitancy may be a barrier to restarting clinical research in PLWH [61, 62]. Reasons for hesitancy should be explored and the protective effect of vaccination, including reassuring vaccine safety and efficacy data, shared. We recommend this should be adapted according to the setting, using data specific to the nationally available vaccines, explaining in particular the risks and benefits of enrolling into HIV “cure” trials in this context. Community engagement will be a key component in providing education on the importance of vaccination to reduce the risk of COVID‐19 infection.

The risks and benefits of study enrolment and/or continuing in a study for a participant not willing to receive an available COVID‐19 vaccine must be carefully assessed. For trials where such a risk–benefit assessment indicates it is appropriate to proceed, there must be a robust informed consent process that explicitly includes information about the increased risks of enrolment in a trial with increased risks of COVID‐19 exposure.

### 2.4 Study implementation and management

Detailed implementation and recommendations for how to safely continue with HIV clinical research during the ongoing SARS‐CoV‐2 pandemic are described on the IAS website [45]. In summary, practical considerations should include: use of telemedicine where practical and feasible; provision of regular SARS‐CoV‐2 testing, ideally PCR; limitation of movement of study participants, in particular during local surges of cases; and access to routinely available SARS‐CoV‐2 prophylactic vaccinations wherever possible, prior to or during enrolment into an HIV clinical trial [63, 64].

#### 2.4.1 SARS‐CoV‐2 testing

SARS‐CoV‐2 testing should be offered regularly throughout the study. Where available, PCR testing is recommended. An antigen test may be appropriate depending on the complexity of the study and whether this includes an ATI, local factors, including availability of tests, infection prevalence and speed of result [65]. While asymptomatic SAR‐CoV‐2 antigen testing has a lower sensitivity than PCR (ranging from 52.5% to 69.9%) [66, 67], high specificity and negative predictive value (99.6% and 97.2%, respectively) [67] could adequately screen asymptomatic carriers in areas where PCR testing is not routinely available.

### 2.5 The unknowns

There remain multiple unknown aspects of the interaction between HIV and SARS‐CoV‐2 infection that may be incorporated into future mitigation strategies as data become available. These include:

- The extent to which severe HIV‐associated immunosuppression or uncontrolled HIV viraemia determines COVID‐19 outcomes
- The theoretical effect of COVID‐19‐related systemic inflammation on non‐AIDS‐related mortality
- SARS‐CoV‐2 vaccine immunogenicity and durability in PLWH
- Risk of “long‐COVID” among PLWH

## 3 CONCLUSIONS

The highly dynamic nature of the COVID‐19 pandemic will likely continue to pose challenges to HIV research. However, to enable ongoing safe research to proceed for PLWH, it is key that rational, ethical and pragmatic approaches are adopted. To facilitate this, close partnerships with communities, public health authorities and researchers will be critical, as well as flexibility in study implementation. Ultimately, we have to anticipate that SARS‐CoV‐2 will become endemic globally and booster dosing schedules will be recommended long term. COVID‐19 prevention and risk mitigation strategies will remain a key consideration when implementing future HIV clinical trials.

We hope this document will provide guidance to safely and optimally perform HIV‐related clinical trials involving PLWH.

**Steering group Members**

**Table:** 

Member	Institution
Sharon Lewin	The Peter Doherty Institute for Infection and Immunity, The University of Melbourne and Royal Melbourne Hospital, Melbourne, Australia
Steve Deeks	University of California, San Francisco (UCSF), San Francisco, USA
Beatriz Mothe	IrsiCaixa AIDS Research Institute, HUGTIP, Badalona, Spain
Sarah Fidler	Department of Infectious Disease, Imperial College London, London, UK
Ian Sanne	University of the Witwatersrand, Johannesburg, South Africa
Beatriz Grinsztejn	Fundação Oswaldo Cruz. Rio de Janeiro, Brazil
Maureen Luba	AVAC, Lilongwe, Malawi
Simon Collins	i‐Base, London, UK
Bridget Haire	Kirby Institute, Sydney, Australia

## COMPETING INTERESTS

The authors have declared no competing interests.

## AUTHORS CONTRIBUTIONS

MH and SF drafted the first version of the manuscript. All authors participated in rounds of discussion and contributed to writing and editing of the manuscript.

## Data Availability

Data sharing is not applicable to this article as no new data were created or analyzed.
